# Protocol to study social transmission of stress in group-housed mice using restraint stress

**DOI:** 10.1016/j.xpro.2025.104224

**Published:** 2025-11-27

**Authors:** Dennis Mink, Dennis Horvath, Michael Basler

**Affiliations:** 1 Institute of Cell Biology and Immunology Thurgau (BITG) at the University of Konstanz, 8280 Kreuzlingen, Switzerland; 2 Division of Immunology, Department of Biology, University of Konstanz, 78457 Konstanz, Germany; 3 Centre for the Advanced Study of Collective Behaviour, University of Konstanz, 78457 Konstanz, Germany

**Keywords:** Health Sciences, Immunology, Model Organisms

## Abstract

Here, we present a protocol to study social transmission of stress in group-housed mice using restraint stress. We describe steps for exposing mice to restrained conspecifics separated by an acrylic divider to induce indirect stress responses. We detail procedures for acute and chronic paradigms, followed by behavioral, physiological, and immunological assessments in both restrained and bystander mice. The protocol allows for the investigation of sensory and social mechanisms underlying stress contagion, while excluding visual cues.

For complete details on the use and execution of this protocol, please refer to Horvath et al.^1^

## Highlights

• Protocol for inducing social stress transmission in group-housed mice
• Guidance on performing acute and chronic restraint stress paradigms
• Steps for collection of blood, organs, and lymphoid tissues
• Assessment of behavioral, physiological, and immunological responses

## Abstract (editor highlights)

Publisher’s note: Undertaking any experimental protocol requires adherence to local institutional guidelines for laboratory safety and ethics.

## Graphical abstract

**Figure:**
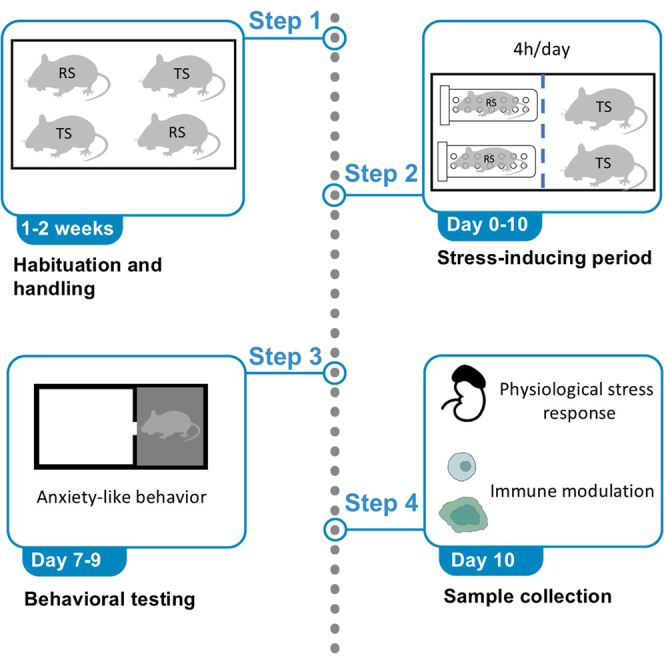

## Before you begin

### Innovation

This protocol provides a controlled and reproducible approach to study the social transmission of stress in group-housed mice while excluding visual cues between animals. This design distinguishes it from existing observer models (e.g., vicarious social defeat stress), where rodents observe a conspecific experiencing pain or fear.^2^^,^^3^ By physically separating mice with an acrylic divider that blocks vision but allows olfactory, auditory, and social cues after the stress procedure, this method enables investigation of how non-visual cues contribute to stress contagion. Although previous observer paradigms often allow visual exposure to a stressed conspecific, the contribution of visual stimuli to stress contagion in mice is likely minor compared to non-visual sensory signals.^4^

The protocol integrates acute and chronic stress paradigms and allows the combined evaluation of behavioral, physiological, and immunological outcomes in both directly and indirectly stressed mice. It is compatible with a wide range of downstream analyses, including immunological analyses, physiological assessments, and behavioral testing for anxiety- or social-related phenotypes.

By providing a standardized and easily implemented setup, this method facilitates reproducible research on the mechanisms underlying social stress transmission and its consequences for social and immune regulation in rodents.

### Institutional permissions

All experiments must be conducted in strict accordance with applicable laws, regulations, and internationally recognized ethical guidelines. All experimental procedures described herein were approved by Regierungspräsidium Freiburg (G-20/113, G-22/075, and G-24/046) (Germany).

### Acclimatization and habituation period

**Timing: 1–2 weeks**

1. Recruit a group of animals with minimal age gap, preferentially littermates to reduce general stress.

***Note:*** For male mice, only use littermates. We generally used C57BL/6 mice between 8–12 weeks of age for our studies.

2. House 4 mice per cage with free access to food and water in a quiet room with controlled temperature and humidity and a split light-dark cycle.

***Note:*** For example, light on at 7 am; light off at 7 pm.

**CRITICAL:** While the number of mice per cage can be changed, this might have considerable effects on the dynamics of stress transmission. We always worked with 4 mice per cage containing 2 directly stressed and 2 indirectly stressed mice. Changing the ratio of directly stressed to indirectly stressed mice might modulate the extent of stress transmission.

**CRITICAL:** This protocol is compatible with both male and female mice. However, male mice must be group-housed from birth to avoid aggressive interactions, which can occur when unfamiliar males are introduced. In contrast, we observed no issues when grouping previously unfamiliar female mice.

3. Conduct daily non-aversive handling of the mice to habituate them to human interaction, which minimizes stress responses to handling during the final experiment.

***Optional:*** Monitoring of stress hormones can be performed before the beginning of the stress procedure to obtain baseline levels of hormones.

***Note:*** For example, by submandibular vein puncture or tail vein puncture 24 hours before beginning of the stress procedure.

***Note:*** Collect blood at the same circadian time you will use for post-stress sampling.

***Note:*** Also monitoring the establishment of social hierarchies during this phase might provide valuable insight.

## Key resources table

**Table:** 

REAGENT or RESOURCE	SOURCE	IDENTIFIER
**Chemicals, peptides, and recombinant proteins**		

Albumin from chicken egg white	Merck	Cat#A2512-1G
OVA (257-264) (H-2Kb) (SIINFEKL)	peptides & elephants	Cat# EP01994

**Critical commercial assays**		

Corticosterone ELISA kit	Enzo Life Sciences	Cat#ADI-900-097
Mouse IL-6 ELISA Ready-SET-Go! kit	Invitrogen eBioscience	Cat#12364003
Mouse TNF ELISA Ready-SET-Go! kit	Invitrogen eBioscience	Cat#15591107
LEGENDplex mouse anti-virus response panel (13-plex)	BioLegend	Cat#740621

**Experimental models: Organisms/strains**		

C57BL/6 mice, aged 8–12 weeks, male and female	Charles River	N/A

**Software and algorithms**		

EthoVision XT 15	Noldus	N/A
Prism	GraphPad	N/A
SPSS Statistics v.29.01	IBM	N/A

**Other**		

Lipopolysaccharides from *Escherichia coli* O111:B4	Sigma	Cat# L2630

## Materials and equipment

### Restraint tubes

**Figure 1:**
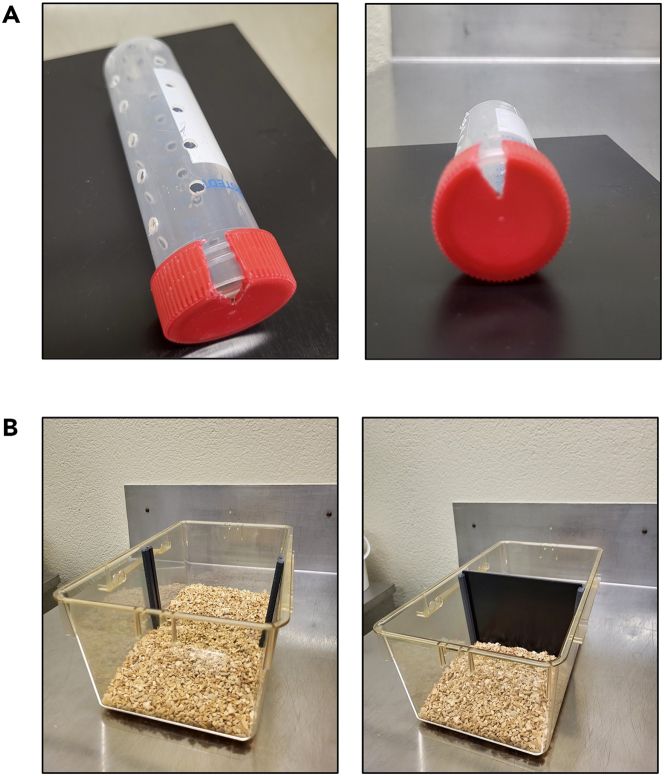
Restraint tubes and cage dividers (A) Restraint tubes are prepared by drilling approx. 30–40 holes into 50 mL conical tubes to ensure adequate airflow during restraint. A small section of the lid is cut to allow the mouse’s tail to pass through. (B) In standard mouse cages, holders were installed to insert the cage divider made of acrylic glass.

Mouse restrainers are commercially available (e.g., Cat# Z756903, Merck). Alternatively, a custom restraining device can be constructed using a 50-mL conical tube. We constructed tubes by drilling (approx. 30–40 holes per tube). Ensure that all holes have smooth edges to prevent injury to the animals (Figure 1A).

### Cage dividers

To spatially and visually isolate directly stressed mice (restraint stress, RS) from indirectly stressed (stress transmission, TS) mice during the restraint procedure, an opaque cage divider made of acrylic glass is inserted. This prevents physical and visual contact during restraint stress procedure. Measure cage dimensions carefully (height, width, depth) of the standard cage you use and cut a divider out of acrylic glass sheets, the height should be just below the cage lid and width tight enough to prevent movement (Figure 1B).

**CRITICAL:** Do not use transparent materials, visual separation is essential to reduce direct behavioral cues during the restraint stress procedure. Ensure tight fit to avoid movement of the restraint tubes during animal activity.

## Step-by-step method details

### Stress-inducing period

**Timing: 1 day (acute) to 10 days (chronic)**

Physical restraint is a widely used method to induce stress in animal models for studying immune and physiological responses.^5^^,^^6^ In this approach, mice are placed in tubes with ventilation holes that limit movement without causing pain, constriction, or suffocation.^7^ Depending on how often this procedure is repeated, it can be used to study acute or chronic stress.^8^^,^^9^

1. Before starting the stress procedure, assign each cage of mice either to a stress group or control group.

Inside the stress cages, assign each mouse either to the directly stressed (RS) or indirectly stressed group (TS).

***Note:*** Each mouse should be uniquely marked to enable unambiguous individual identification.

**CRITICAL:** Animal stress levels can be influenced by numerous confounding factors, including environmental noise, vibration, fluctuating room temperatures and interaction with experimenters or animal care takers. To account for this variability, it is essential to include unstressed control groups in every experiment to establish baseline stress levels and to detect any deviations from these.

**Figure 2:**
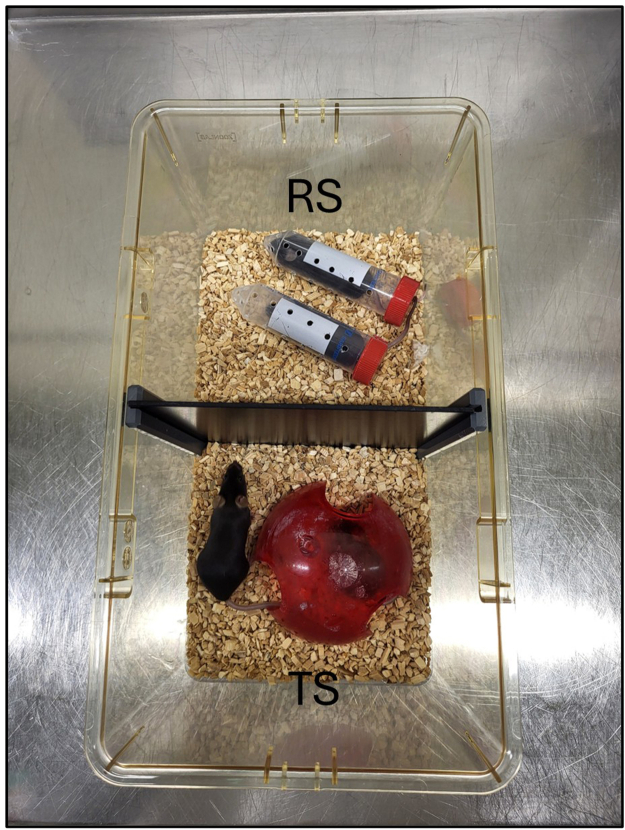
Experimental setup with cage divider RS mice are placed in restraint tubes (top), while TS mice move freely (bottom). After 4 hours, RS mice are released from the tubes, the dividers are removed, and all mice interact freely for 20 hours until the next procedure.

***Note:*** The mice belonging to the TS group have access to food and water during the procedure.

***Note:*** For the control group, place two mice on each side of the divided cage. No stress is applied to these four mice.

2. Record the weight of each mouse before the start of the stress procedure.
3. Take out all mice from the home cage and gently put them into a separate cage.
4. Insert the “Cage Divider”.
5. Restrain two mice into restraint tubes (one mouse per tube) and place the two tubes back into one side of the divided cage. Place the other two mice on the other side. (Methods video S1) (Figure 2).

6. After four hours, free the restrained mice and remove the divider.

**CRITICAL:** Mice can also be separated into different cages for the stress procedure; however, we found that this induces considerable stress also in control mice.

**CRITICAL:** Upon returning to their home cage, we found that especially male mice, when placed in different cages, showed overly aggressive behavior towards each other.

**CRITICAL:** Any additional procedure, such as taking blood, injection of substances etc. might affect the state of the mouse leading to a stress response. We recommend limiting such procedures as much as possible and treating control and stressed groups in the very same way.

***Optional:*** The duration of restraint stress can be varied depending on the magnitude of stress that is desired. We found that 4 hours was enough to induce massive physiological stress responses in directly stressed mice.^1^

***Optional:*** To assess the impact of direct stress and stress transmission on adaptive immunity, we performed immunizations on day 4 of the stress procedure. Our immunization consisted of an s.c. injection of 5 mg poly(lactic-co-glycolic acid) (PLGA)-microparticles containing the model antigen ovalbumin together with the adjuvant Riboxxim. Previous studies have shown that T cell responses are impaired only when stress precedes infection or immunization, but not when stress is applied concurrently.^10^

7. Apply the stress procedure once (acute stress) or repeat it daily (chronic stress) for up to 10 days.

***Note:*** We have not tested longer stress procedures, however, prolonged restraint stress might lead to habituation.^11^

***Note:*** Stress readouts can be assessed during the stress-inducing period or at the end. We assessed behavioral readouts starting from day 7 of the stress procedure, with one test per day. We performed novel object recognition tests on day 7, open field test on day 8 and the light/dark box test on day 9.

8. After 1 day or 10 days of stress, sacrifice the mice for tissue collection.

Methods video S1. Placement of a mouse into a restrainer: Demonstration of safe handling technique for positioning a mouse in a 50 mL tube, related to step 5

### Collection of samples

**Timing: ∼1–2 h (depending on the number of animals)**

This section describes procedures for collecting blood, organs, and lymphoid tissues after completion of the stress or stress transmission paradigm. Samples should be collected at a consistent time of day (e.g., 2–3 h after lights on) to control for circadian effects on corticosterone and immune parameters.

9. Euthanize mice using CO_2_ asphyxiation followed by cervical dislocation, in accordance with institutional ethical guidelines.

***Note:*** Perform euthanasia in a quiet room to avoid pre-terminal stress elevation.

10. Collect blood via cardiac puncture immediately after euthanasia using a 1 mL syringe with a 25–27G needle.
11. Transfer blood into EDTA- or heparin-coated microcentrifuge tubes.
12. Centrifuge at 2,000 × g for 10 min at 4°C to separate blood plasma.
13. Aliquot blood plasma and store at −80°C for downstream assays.

***Note:*** For example, corticosterone ELISA.

14. Tissue collection.
  a. Adrenal glands: Dissect and weigh both adrenal glands. Normalize weight to body weight.
    ***Optional:*** Fix in 4% formalin in phosphate buffer (55 mM Na_2_HPO_4_, 12 mM NaH_2_PO_4_) for histology.
  b. Thymus and spleen: Remove, weigh, and place in PBS on ice for downstream cell isolation.
    ***Note:*** For example, flow cytometry or cytokine assays.
    ***Note:*** Prepare single-cell suspensions from spleen or thymus for flow cytometry or in vitro stimulation with, for example, LPS.
    ***Optional:*** For studies with neural endpoints, brains can be collected and fixed or snap-frozen for later analysis.
    **CRITICAL:** Complete all dissections quickly and consistently across groups to minimize variability caused by post-mortem degradation or delayed stress hormone clearance.

## Expected outcomes

This protocol induces robust and reproducible physiological, behavioral, and immunological responses in both directly stressed and indirectly stressed (stress-transmission) mice. The extent of these responses depends on the duration (acute vs. chronic) and sex of the animals. Effects after only one session of restraint stress (acute stress) and a 20 h social interaction period were very mild, but chronic stress transmission showed profound effects. This protocol can be combined with other interventions. We applied a vaccination model, however, alternative approaches such as genetic knockout systems or other drug interventions could likewise yield valuable data on the transmission of stress and its consequences. To confirm the induction of direct stress, we found that thymus atrophy was the most consistent and profound readout. In stress transmission mice, adrenal hypertrophy was the most consistent readout.

**Figure 3:**
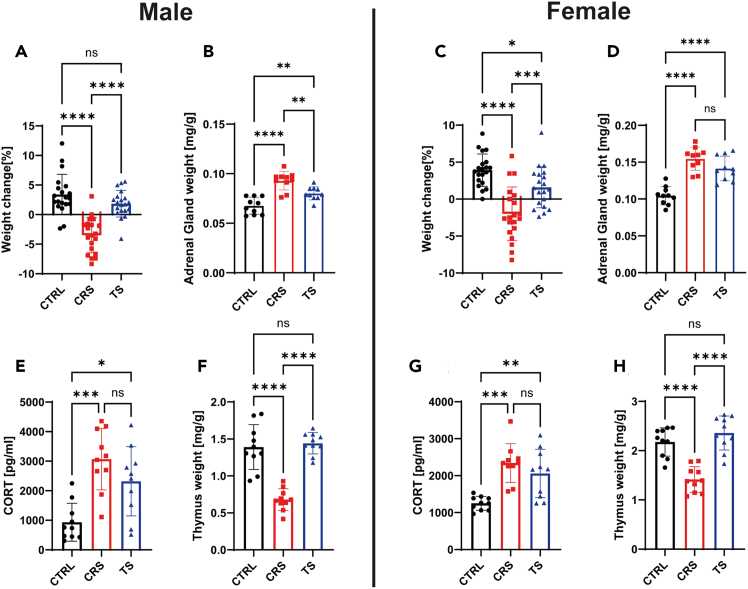
Impacts of chronic stress transmission on physiology Male (n=10 per group) (A) or female (n=10 per group) (C) C57BL/6 mice were subjected to chronic restraint stress (CRS, red squares), stress transmission (TS, blue triangles), or control treatment (CTRL, black dots) and the change in body weight was monitored from day 1 to day 10 of the stress procedure. Male or female C57BL/6 mice (n=10) were subjected to chronic restraint stress (CRS, red squares), stress transmission (TS, blue triangles), or control treatment (CTRL, black dots). One day after the last stress procedure, adrenal glands (B and D) and thymuses (F and H) were harvested and the organ mass was determined relative to the body weight. (E and G) Corticosterone levels were analyzed in the blood plasma via ELISA. Pooled data from three independent experiments are presented as means ± SD. Statistics: ANOVA followed by Tukey’s multiple comparison test; ∗*p* < 0.05, ∗∗*p* < 0.01, ∗∗∗*p* < 0.001, ∗∗∗∗*p* < 0.0001, ns = not significant. (Figure derived from^1^).

Physiologically, both directly stressed and stress-transmission mice showed increased adrenal gland weight, indicating activation of the hypothalamic-pituitary-adrenal (HPA)-axis (Figure 3). Plasma corticosterone levels were significantly increased in chronically stressed and indirectly stressed mice. We found that female mice show greater susceptibility to stress transmission, reflected in impaired weight gain and more pronounced adrenal hypertrophy compared to males. Additionally, thymus weight and size were profoundly reduced in directly stressed mice (Figure 3), a change readily visible to the naked eye.

**Figure 4:**
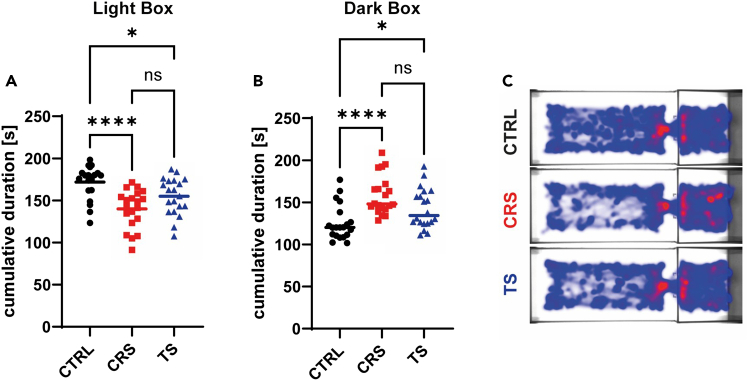
Chronic stress transmission reduces exploratory behavior in mice (A and B) Male (n=10 per group) and female (n=10 per group) C57BL/6 mice were subjected to chronic restraint stress (CRS, red squares), stress transmission (TS, blue triangles), or control treatment (CTRL, black dots) for 10 consecutive days. On day 10 of the stress procedure, mice were placed in a light-dark box arena and tested for exploratory behavior for 5 minutes. The cumulative duration spent in the light box (A), or dark box (B) was quantified by automated video tracking. (C) Merged trials per group of one representative experiment are shown as heatmaps. Pooled data of five independent experiments are presented as means. Statistical significance was determined by one-way ANOVA followed by Tukey’s multiple comparison test; ∗*p* < 0.05, and ∗∗∗∗*p* < 0.0001; ns, not significant. (Figure derived from^1^).

On the behavioral level, stress transmission led to increased anxiety-like behavior, particularly in light-dark box tests, although effects are less prominent than in directly stressed animals (Figure 4). Novel object recognition tests revealed no change in cognition for both directly stressed and stress transmission mice.

**Figure 5:**
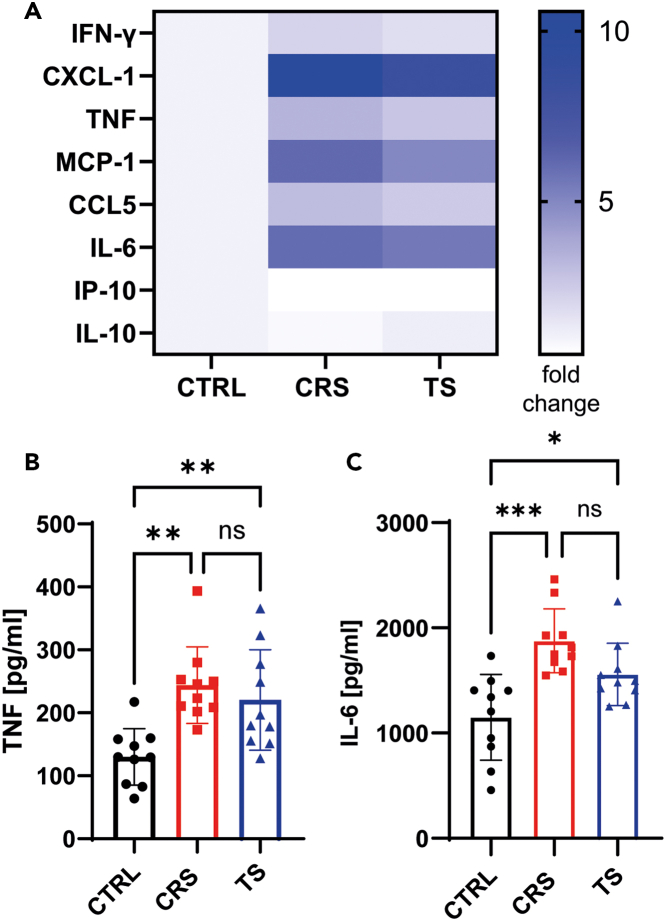
Chronic stress transmission induces elevated cytokine secretion upon LPS stimulation Male (n=5 per group) and female (n=5 per group) C57BL/6 mice were subjected to chronic restraint stress, stress transmission, or control treatment for 10 consecutive days. On day 11, splenocytes were harvested and stimulated in vitro for 75 hours with LPS. Supernatants were screened for cytokine secretion using a flow cytometry based multiplex assay (A). Pooled data of two independent experiments are presented as heatmap plot of fold change relative to controls. TNF (B) or IL-6 (C) levels in the supernatants of chronically restraint stressed (CRS, red squares, n=10) mice or mice subjected to stress transmission (TS, blue triangles, n=10) or control treated mice (CTRL, black dots, n=10) were quantified by ELISA. Data of three independent experiments are presented as means ± SD. Statistical significance was analyzed by one-way ANOVA followed by Tukey’s multiple comparison test; ∗*p* < 0.05, ∗∗*p* < 0.01, ∗∗∗*p* < 0.001, ns = not significant. (Figure derived from^1^).

For immunological outcomes, stress transmission induced an enhanced secretion of pro-inflammatory cytokines (e.g., TNF and IL-6) by splenocytes upon stimulation with 1 μg/ml lipopolysaccharide (LPS) for 75 hours, reaching levels similar to directly stressed mice (Figure 5). Antigen-specific T and B cell responses to a model-antigen vaccination remained largely unaffected in stress-transmission mice, as assessed via intracellular cytokine staining by restimulation of splenocytes with SIINFEKL peptide, in contrast to significantly impaired responses in directly stressed mice.^1^ CD4^+^CD8^+^ T cells in the thymus are sensitive to glucocorticoids.^12^ The number of these cells was markedly decreased in directly stressed mice but not stress transmission mice.^1^

Researchers may extend this model to additional readouts, including neuroendocrine markers (e.g., c-Fos expression), behavioral assays assessing social interaction, anhedonia, or anxiety, as well as transcriptomic or epigenetic profiling to identify systemic stress signatures. Metabolic measures such as glucose tolerance, food intake, and energy expenditure, or microbiome analyses of fecal samples, can further broaden the understanding of systemic stress responses. Longitudinal follow-up studies may also reveal the persistence or reversibility of stress-induced changes.

Overall, this model thus offers a flexible framework to assess stress transmission and to explore its diverse consequences on multiple biological systems.

## Limitations

While this protocol provides a robust and reproducible model to study stress transmission in group-housed mice, several limitations should be considered. First, the method relies on restraint stress as the sole mode of inducing the primary stress response. Although this approach offers high reproducibility and avoids physical injury, unlike aggression-based paradigms, it does not fully replicate the diversity of psychosocial stressors that mice may encounter in more naturalistic or socially complex environments.

Additionally, the model assumes that stress transmission occurs uniformly across cohoused individuals, but the actual mechanisms remain poorly understood. It remains unclear whether olfactory, auditory, or social cues drive the observed effects. In contrast to many other stress contagion models, our design prevents direct visual observation of stressed mice. Nonetheless, factors such as individual susceptibility, social hierarchy, and buffering behaviors could significantly influence outcomes within cages. It is therefore advisable to monitor social dynamics or dominance status, particularly in male mice, to account for potential variability.

## Troubleshooting

### Problem 1

Aggressive behavior, especially in male mice, leading to injured animals or induction of stress even in control mice.

### Potential solution

Aggression at the cage level is a multifaceted phenomenon, for which no established solutions currently exist.^13^ However, it may be mitigated by: i) establishing stable groups before the experiment, by keeping siblings or familiar mice together; ii) transferring the bedding during cage changes; iii) keeping the ambient temperature at 20°C–22°C, while providing abundant nesting materials; iv) avoiding exposure to female scent that can trigger aggression,^14^ for instance through housing male and female mice in separate rooms.

While this protocol aims to prevent aggression by keeping mice in their home cages during the stress procedure, it is still possible that you may observe increased fighting between mice after removing the cage divider. To avoid territory invasion between the mice it is possible to increase the dimension of your cage used. Always ensure that male mice are already familiar with each other since birth to avoid aggression. If aggression is too severe and wounding occurs, the specific cages need to be excluded from the experiments.

### Problem 2

High variations in levels of corticosterone.

### Potential solution

It is very important to control the time point of blood collection, since the levels of corticosterone follow a circadian rhythm. It is also important to preserve serum quality by processing blood samples promptly after collection.

### Problem 3

High variability in stress transmission group.

### Potential solution

We observed that some mice exhibit stronger physiological responses to stress transmission than others. Although the underlying cause remains unclear, this variability presents an interesting approach for future investigation. Baseline corticosterone or oxytocin levels, as well as social hierarchy dynamics, may serve as potential predictors of susceptibility to stress transmission.

### Problem 4

Control mice exhibit physiological stress response or large variations in stress responses between experiments.

### Potential solution

We found that even minor environmental changes can inadvertently induce stress in control animals, leading to physiological stress responses or variability across experiments. To minimize this, it is essential to maintain highly consistent experimental conditions, including temperature, humidity, light cycles, cage handling routines, and noise levels, throughout the study. For example, we noted substantial fluctuations in stress levels during periods of construction in a nearby building or on other floors of the same building. Avoid introducing new handlers or altering room conditions and ensure that all groups are treated identically apart from the intended stress exposure.

### Problem 5

Mice exhibit unusual behaviors during behavioral testing such as light/dark box or open field test.

### Potential solution

Acclimate mice to the behavioral testing room for at least 1–2 hours before the start of testing to minimize novelty-induced stress and variability. Ensure the testing environment is quiet, stable, and free from strong odors or disturbances that could influence behavior. Male and female mice should be tested using separate apparatuses whenever possible. If shared equipment is used, it must be thoroughly cleaned and ventilated between sessions to minimize sex-specific olfactory influences.

## Resource availability

### Lead contact

Further information and requests for resources and reagents should be directed to and will be fulfilled by the lead contact, Michael Basler (michael.basler@bitg.ch).

### Technical contact

Technical questions on executing this protocol should be directed to and will be answered by the technical contact, Dennis Mink (dennis.mink@uni-konstanz.de).

### Materials availability

This study did not generate new unique reagents.

### Data and code availability

• All data reported in this paper will be shared by the lead contact upon request.
• This paper does not report new code.
• Any additional information required to reanalyze the data reported in this paper is available from the lead contact upon request.

## Acknowledgments

We thank the animal research center (TFA) of the University of Konstanz for mouse breeding.

## Author contributions

D.M., conceptualization, methodology, validation, formal analysis, investigation, and visualization; D.H., conceptualization, methodology, validation, formal analysis, investigation, and writing; M.B., validation, supervision, and writing – reviewing and editing.

## Declaration of interests

The authors declare no competing interests.
